# Double-negative T cells ameliorate psoriasis by selectively inhibiting IL-17A-producing γδ^low^ T cells

**DOI:** 10.1186/s12967-024-05132-8

**Published:** 2024-04-02

**Authors:** Yunxiong Wei, Guangyong Sun, Yang Yang, Mingyang Li, Shimeng Zheng, Xiyu Wang, Xinjie Zhong, Zihan Zhang, Xiaotong Han, Haiyan Cheng, Dong Zhang, Xueling Mei

**Affiliations:** 1 Immunology Research Center for Oral and Systemic Health, Beijing Friendship Hospital, Capital Medical University, Beijing, 100050 China; 2 Medical Research Center, Beijing Institute of Respiratory Medicine and Beijing Chao-Yang Hospital, Capital Medical University, Beijing, China; 3 Department of Gastroenterology, Beijing Chao-Yang Hospital, Capital Medical University, Beijing, 100020 China; 4 Beijing Laboratory of Oral Health, Capital Medical University School of Basic Medicine, Beijing, 100069 China; 5 Department of Dermatology, Beijing Friendship Hospital, Capital Medical University, Beijing, 100050 China

**Keywords:** Psoriasis, γδ T cells, Double Negative T cells, IL-17A, Cell therapy

## Abstract

**Background:**

Psoriasis is a chronic immune-mediated skin condition. Although biologic treatments are effective in controlling psoriasis, some patients do not respond or lose response to these therapies. Thus, new strategies for psoriasis treatment are still urgently needed. Double-negative T cells (DNT) play a significant immunoregulatory role in autoimmune diseases. In this study, we aimed to evaluate the protective effect of DNT in psoriasis and explore the underlying mechanism.

**Methods:**

We conducted a single adoptive transfer of DNT into an imiquimod (IMQ)-induced psoriasis mouse model through tail vein injection. The skin inflammation and IL-17A producing γδ T cells were evaluated.

**Results:**

DNT administration significantly reduced the inflammatory response in mouse skin, characterized by decreased skin folds, scales, and red patches. After DNT treatment, the secretion of IL-17A by RORc^+^ γδ^low^ T cells in the skin was selectively suppressed, resulting in an amelioration of skin inflammation. Transcriptomic data suggested heightened expression of NKG2D ligands in γδ^low^ T cells within the mouse model of psoriasis induced by IMQ. When blocking the NKG2D ligand and NKG2D (expressed by DNT) interaction, the cytotoxic efficacy of DNT against RORc^+^IL17A^+^ γδ^low^ T cells was attenuated. Using *Ccr5*^−/−^ DNT for treatment yielded evidence that DNT migrates into inflamed skin tissue and fails to protect IMQ-induced skin lesions.

**Conclusions:**

DNT could migrate to inflamed skin tissue through CCR5, selectively inhibit IL-17-producing γδ^low^ T cells and finally ameliorate mouse psoriasis. Our study provides feasibility for using immune cell therapy for the prevention and treatment of psoriasis in the clinic.

**Supplementary Information:**

The online version contains supplementary material available at 10.1186/s12967-024-05132-8.

## Background

Psoriasis is a chronic, immune-mediated systemic dermatosis affected by genetic and multiple environmental factors. It has been recognized as a public health burden and is estimated to affect ~ 125 million people worldwide [1, 2]. Immunological and genetic studies have identified the IL-17/IL-23 axis as a key driver of psoriasis pathogenesis [3–5].

Although γδ T cells are only a small portion of total T cells, existing evidence suggests that aberrantly activated γδ T cells play a pivotal role in the pathogenesis of psoriasis [6–8]. IL-23 predominantly stimulates dermal γδ T cells to produce IL-17, which leads to disease progression. Dermal γδ T cells constitutively express IL-23 receptor (IL-23R) and the transcription factor RORγt [9]. In psoriasis patients, γδ T cells are greatly increased in affected skin and produce large amounts of IL-17 [9]. We also previously demonstrated that significantly expanded γδ T cells with high glycolysis levels drive psoriasis development in imiquimod (IMQ)-treated mice [10]. Therefore, γδ T cells have gained considerable attention as an attractive target for psoriasis immunotherapy.

Double-negative T cells (DNT), which are defined as TCRαβ^+^CD3^+^CD4^−^CD8^−^NK1.1^−^ (mouse)/CD56^−^ (human), are essential for maintaining immune system homeostasis with antigen specificity [11, 12]. We have shown that DNT could prevent or reverse the onset of type 1 diabetes, allergic asthma, transplant rejection and the development and progression of nonalcoholic steatohepatitis [13–15]. However, whether adoptive transfer of DNT could improve the immune microenvironment of skin tissue and alleviate the pathogenesis of psoriasis remains unclear.

In this study, we demonstrated that adoptive transfer of DNT could alleviate the pathogenesis of psoriasis by downregulating the inflammatory response in mouse models. DNT can migrate to inflamed skin and draining lymph nodes and selectively suppress IL-17-producing γδ T cells through NKG2D signaling. Our findings provide a novel immune cell therapy for psoriasis.

## Methods

### Mice

*Rorc*^*IRES−tdTomato−T2A−Cre*^ mice were generated by Shanghai Model Organisms Center, Inc. using a CRISPR/Cas9-based approach. Briefly, a targeting vector containing the following components was constructed: IRES-tdTomato-T2A-Cre. Cas9 mRNA was transcribed in vitro with the mMESSAGE mMACHINET7 Ultra Kit (Ambion, TX, USA) according to the manufacturer’s instructions and subsequently purified using the MEGAclear™ Kit (Thermo Fisher, USA). 5'-GGGCTGTCAAAGTGATCTGG-3' was chosen as the Cas9 targeted guide RNA (sgRNA), transcribed in vitro using the MEGAshortscript Kit (Thermo Fisher, USA) and subsequently purified using the MEGAclear™ Kit. The donor vector with sgRNA and Cas9 mRNA was microinjected into fertilized C57BL/6 J eggs. F0 generation mice positive for homologous recombination were identified by tail genomic DNA PCR.

*Ccr5*^*−/−*^(B6.129P2-Ccr5^tm1Kuz^/J), B6 CD45.1 (B6. SJL-*Ptprc*^*a*^* Pepc*^*b*^/BoyJ) were purchased from the Jackson Laboratory (ME, USA). Wild-type C57BL/6 mice were purchased from HFK Laboratory (Beijing, China). The mice were accommodated in a sterile environment free from pathogens, with a pleasant temperature setting and a 12-h light/dark cycle.

### Animal models

Mice underwent shaving, followed by topical application of either 62.5 mg of IMQ cream (3 M, USA) or vehicle cream (control) on their backs for six consecutive days. On Day 7, the mice were sacrificed for further analysis. All experiments involving animals were conducted in adherence to the ethical principles for animal research and received approval from the Animal Ethics Committee of Beijing Friendship Hospital, Capital Medical University.

### Generation of DNT and adoptive transfer

The DNT were generated and expanded in vitro as previously established protocols [16]. CD3^+^TCRβ^+^CD4^−^CD8^−^NK1.1^−^ DNT were sorted using a FACSAria II sorter (BD Biosciences, USA). Finally, 5 × 10^6^ DNT were intravenously adoptively transferred into mice through tail vein injection.

### Psoriasis area and severity index (PASI) assessment

The severity of the lesion following IMQ exposure was assessed using the PASI score. The score ranged from 0 to 4 based on the erythema, scale, and thickness of the skin, where 0 indicated normal skin and 4 indicated a very severe alteration. The PASI score was evaluated daily for each mouse, and the assessment was conducted in a blinded manner.

### Cytotoxicity assay and anti-NKG2D blocking antibody administration in vitro

CD45.1^+^ γδ T cells were obtained from the spleens and peripheral lymph nodes according to the protocol provided by the EasySep™ Release Mouse PE Positive Selection Kit (StemCell Technologies Inc., Canada) and cocultured with CD45.2^+^ DNT (1:1) in a round-bottom 96-well plate for 24 h. Annexin V and caspase-3 staining were performed to detect the apoptosis of CD45.1-positive cells via flow cytometry.

Regarding the anti-NKG2D blocking assay, CD45.2^+^ DNT were preincubated with an anti-NKG2D antibody for 1 h before being cocultured with CD45.1^+^ γδ T cells. After 24 h of coculture, Annexin V and caspase-3 staining were performed to detect the apoptosis of CD45.1-positive cells via flow cytometry.

### Isolation of murine leukocytes from skin tissues

To isolate leukocytes from skin tissue, the diseased skin region was first removed from the mouse's back and subsequently cut into very small pieces. The tissue was then digested in 3.5 ml of RPMI medium (GIBCO, USA) containing 0.1% BSA, 15 mM HEPES, 1 mg/mL collagenase D (Roche), and 450 μg/mL Liberase TL (Roche) for 60 min at 37 °C in a shaking incubator at 200 rpm. After digestion, the tissue was further disrupted by passing through an 18 G needle to release cells. The cell suspension was then filtered through a 70 μm cell strainer, and the digestion was halted by adding ice-cold D-PBS containing 0.1% BSA (Sigma‒Aldrich, USA) and 5 mM EDTA (Invitrogen). To enrich for lymphocytes, a three-layer density gradient using Percoll (GE Healthcare) was employed. The cells obtained from the digestion were carefully layered on top of the 40% and 70% Percoll layers and centrifuged for 20 min at 2000 rpm without braking. Cells at the interface between the 40% and 70% Percoll layers were collected for subsequent analysis.

### Flow cytometry

Flow cytometry analysis was conducted using a BD Aria II flow cytometer, and the data were analyzed using FlowJo software. Antibodies against TCRγδ (GL3), TCRβ (H57-597), CD3 (17A2), CD4 (GK1.5), CD8 (53–6.7), NK1.1 (S17016D), Annexin V, IFN-γ (XMG1.2), TNF-α (MP6-XT22), IL-17A (TC11-18H10), NKG2D (CX5), and CD45.1 (A20) were purchased from Biolegend. Antibodies against MICAL1 and MICAL2 were purchased from Thermo Fisher.

### RNA sequencing and analysis

Skin-draining lymph nodes (LNs) and skin were obtained from heathy and psoriatic mice with or without DNT therapy and underwent sequencing using the standard Illumina protocol provided by Annoroad Gene Technology, Beijing. Each sample represented γδ T cells obtained from 15 mice. The sequencing reads were aligned to the mouse genome (Mm9) using HISAT2, and gene counts were estimated using HTSeq. Differential gene expression analysis was performed using the R package DESeq2 to identify genes that were differentially expressed (DEGs). Genes with a fold-change ≥ 1.5 and a P value < 0.05 were considered DEGs. Subsequently, the R package clusterprofiler was utilized to conduct Gene Ontology (GO) enrichment analysis and Kyoto Encyclopedia of Genes and Genomes (KEGG) of the DEGs. Gene set enrichment analysis (GSEA) was performed using the tools provided by OmicStudio (https://www.omicstudio.cn/tool). To facilitate data sharing and reproducibility, the results have been deposited in the Gene Expression Omnibus (GEO) database under accession number GSE248626.

### Real-time PCR

Total RNA was extracted from the infarct area using TRIzol reagent (Sigma‒Aldrich, USA) and converted into cDNA through reverse transcription using PrimeScript™ RT Master Mix (TaKaRa, Japan). Real-time PCR was conducted using the ABI QuantStudio 3 system (Applied Biosystems, USA) following the manufacturer's guidelines. Amplicon expression in each sample was normalized to *Gapdh* expression, and the 2^−ΔΔCt^ method was employed to quantify gene expression. The primers used in the article are listed in Additional file 1: Table S1.

### Statistical analysis

Statistical analyses were conducted using SPSS Statistics (IBM SPSS Statistics for Windows, version 25.0, USA) and Prism 8.0 software (GraphPad Software, USA). Data are presented as the mean ± standard deviation (SD). The normal distribution of variables was assessed using the Shapiro‒Wilk test. Differences between the two groups were compared using the t test for normally distributed variables and the Kruskal‒Wallis test for nonnormally distributed variables. One-way ANOVA with post hoc tests was used for normal variables, while the Kruskal‒Wallis test was employed for nonnormal variables in cases of multiple comparisons. Statistical significance was defined as a two-sided p value of < 0.05 (*p < 0.05, **p < 0.001).

## Results

### Adoptive transfer of DNT alleviated IMQ-induced murine psoriasis

**Fig. 1:**
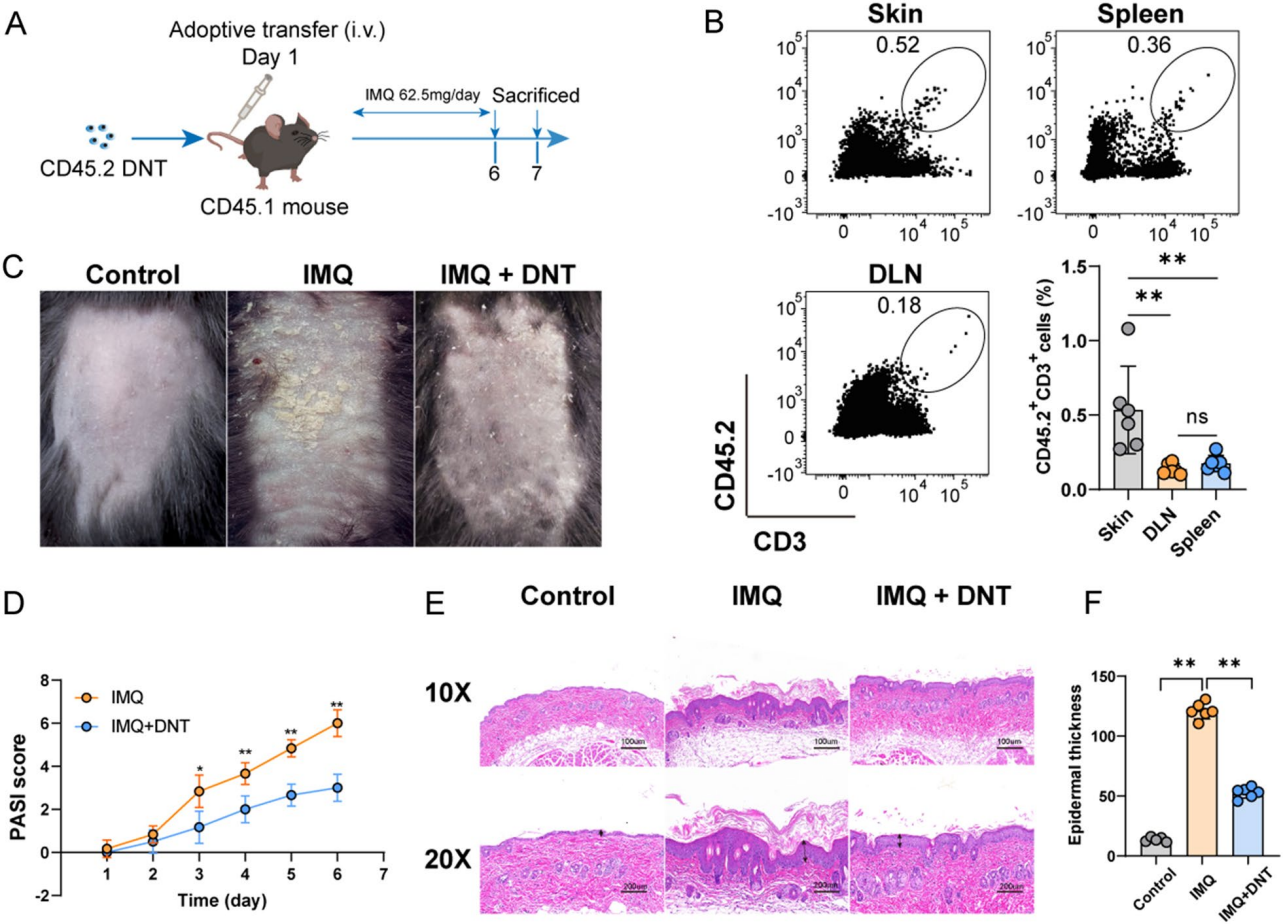
Adoptive transfer of DNT alleviated IMQ-induced murine psoriasis. **A** Flow chart of the adoptive transfer of DNT. **B** The in vivo distribution of transferred DNT in mice and statistical analysis of the proportion of transferred DNT among the skin, LN and spleen. **C** Pictures of control, IMQ-induced psoriasis and DNT-treated mice. **D** Comparison of the PASI scores of psoriatic mice treated with or without transferred DNT after IMQ exposure. **E** Representative H&E staining of skin sections obtained from the control, IMQ-induced psoriasis and DNT-treated mice. The scale bar represents 100 µm and 200 µm. **F** Statistical analysis of the layers of keratinocytes in HE sections obtained from the control, IMQ-induced psoriasis and DNT-treated mice. Data are presented as the mean ± SD; n = 6 mice/group. The normal distribution of variables was assessed using the Shapiro‒Wilk test. One-way ANOVA with a post hoc test for normal variables and the Kruskal–Wallis test for nonnormal variables were used for multiple comparisons. *p < 0.05, **p < 0.01

To explore the protection of DNT in psoriasis, we adoptively transferred DNT through tail vein injection to mice that were started to receive 6 days of IMQ treatment (Fig. 1A). On Day 7, the transferred DNT mainly accumulated within the skin tissues of recipient mice (Fig. 1B). The mice treated with IMQ showed increased skin folds, scales, and red patches. However, DNT alleviated the scaly and erythematous epidermal symptoms among IMQ-treated mice (Fig. 1C). In comparison with mice subjected solely to IMQ exposure, mice treated with DNT exhibited a decrease in PASI scores (Fig. 1D). Histological examination also revealed IMQ-induced psoriasis phenotypes, i.e., increased thickening of the epidermal layer, elongation of rete-like ridges and hyperkeratosis, and inflammatory cell infiltration. Nevertheless, DNT treatment alleviated inflammatory cell infiltration and improved hyperkeratosis in psoriatic mice (Fig. 1E). Moreover, the layers of keratinocytes in the skin were significantly decreased due to DNT treatment (Fig. 1F). Collectively, these findings provide evidence that the administration of DNT effectively alleviated IMQ-induced psoriasis.

### Transcriptome sequencing analysis of skin and DLNs in IMQ-induced murine psoriasis with or without DNT treatment

**Fig. 2:**
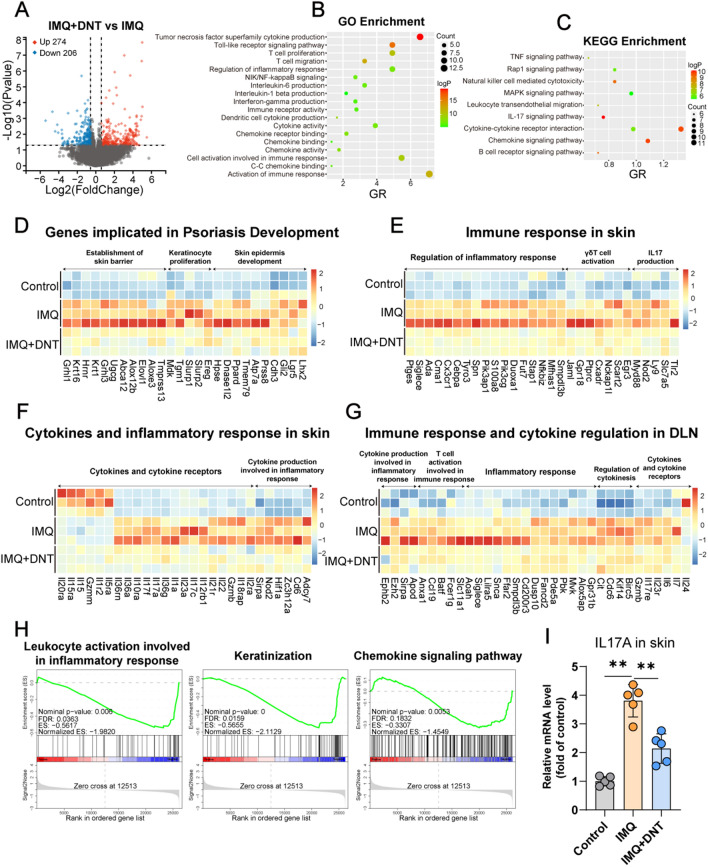
Transcriptome sequencing analysis of skin and DLNs in IMQ-induced murine psoriasis with or without DNT treatment. **A** Distributions of differentially expressed genes with upregulated (274) and downregulated (206) expression in the skin of IMQ-induced murine psoriasis with or without DNT treatment are depicted in a volcano plot. (P value < 0.05 and an absolute fold change ≥ 1.5. n = 3/group). **B** and **C** Biological process and KEGG pathway analyses were performed on the basis of differentially expressed genes with significantly upregulated and downregulated expression in the skin of IMQ-induced murine psoriasis with or without DNT treatment. **D** Heatmap showing the genes associated with psoriasis development in the skin of IMQ-induced murine psoriasis, including the DNT treatment group, psoriasis group, and control group. **E** and **F** Heatmap displaying the genes related to the immune response and inflammatory response in the skin of IMQ-induced murine psoriasis, including DNT treatment, psoriasis, and control groups. **G** Heatmap showing genes related to the immune response and cytokine regulation in the lymph nodes of the DNT treatment, IMQ-induced psoriasis, and control groups. **H** GSEA of leukocyte activation involved in the inflammatory response, keratinization and chemokine signaling pathways in the skin from the DNT treatment and IMQ-induced psoriasis groups. **I** Relative mRNA level of IL17A in skin tissue. Data are presented as the mean ± SD; n = 5 mice/group. The normal distribution of variables was assessed using the Shapiro‒Wilk test. One-way ANOVA with a post hoc test for normal variables and the Kruskal‒Wallis test for nonnormal variables were used for multiple comparisons. *p < 0.05, **p < 0.01

To gain deeper insights into the protective effect of DNT in IMQ-induced psoriasis, we conducted transcriptome sequencing on the skin and skin draining lymph nodes (DLNs) of psoriasis mice treated with or without DNT. The volcano plot analysis indicates that, in comparison to IMQ-induced psoriasis mice, DNT-treated mice’ skin sequencing results exhibit 480 differentially expressed genes (with a P value < 0.05 and an absolute fold change ≥ 1.5) (Fig. 2A). Gene Ontology (GO) and Kyoto Encyclopedia of Genes and Genomes (KEGG) pathway analyses revealed that the differentially expressed genes were involved in a variety of cellular functions, such as cytokine production, T-cell proliferation, cell migration, cytokine activity, regulation of inflammatory response, immune response, and chemokine binding. Notably, the IL-17 signaling pathway, a key pathway in IMQ-induced psoriasis, was also implicated. Furthermore, several signaling pathways were identified, including the nuclear factor kappa B (NF-κB) pathway, TNF signaling pathway, Rap1 signaling pathway, and mitogen-activated protein kinase (MAPK) signaling pathway (Fig. 2B and C).

As shown in Fig. 2D, the genes associated with the development of psoriasis were downregulated significantly after DNT treatment. These DEGs were enriched in crucial processes, including the establishment of the skin barrier (G*rhl1, Krt16, Hrnr, Krt1, Grhl3, Ugcg, Abca12, Alox12b, Elovl1, Aloxe3, and Tmprss13*), keratinocyte proliferation (*Mdk, Tgm1, Slurp1, Slurp2, and Ereg*), and skin epidermis development (*Hpse, Dnase1l2, Ppard, Tmem79, Atp7a, Prss8, Cdh3, Gli2, Lgr5, and Lhx2*). Furthermore, after DNT administration, genes related to immune responses in the skin exhibited decreased activity, encompassing the regulation of inflammatory responses (*Ptges, Siglece, Ada, Cma1, Cx3cr1, Cebpa, Tyro3, Spn, Pik3ap3, S100a8, Pik3cg, Duoxa1, Fut7, Stap1, Nfkbiz, Mfhas1, and Smpdl3b*), γδ T-cell activation (*Jaml, Gpr18, Ptprc, Cxadr, Nckap1l, Scart2, and Egr3*), and IL17 production (*Myd88, Nod2, Ly9, Slc7a5, and Tlr2*) (Fig. 2E). Moreover, genes related to cytokines and inflammatory responses in the skin also changed following DNT treatment. This included alterations in cytokines and cytokine receptors (*Il20ra, Il15ra, Il15, Gzmm, Il1r2, Il5ra, Il36rn, Il36a, Il10ra, Il17f, Il17a, Il36g, Il1a, Il23a, Il17c, Il12rb1, Il21r, Il22, Gzmb, Il18rap, and Il2ra*), as well as modifications in cytokine production involved in inflammatory responses (*Sirpa, Nod2, Hif1a, Zc3h12a, Cd6, and Adcy7*) (Fig. 2F). Additionally, transcriptome sequencing of DLNs revealed that DNT treatment influenced the expression of genes related to immune responses and cytokine regulation, including cytokine production involved in inflammatory responses (*Ephb2, Ezh2, Sirpa, and Apod*), T-cell activation involved in immune responses (*Anxa1, Ccl19, Batf, Fcer1g, and Slc11al*), inflammatory responses (*Aoah, Siglece, Lilra5, Snca, Ffar2, Smpdl3b, Cd200r3, Dusp10, Fancd2, Pde5a, Pbk, Mvk, Alox5ap, and Gpr31b*), regulation of cytokinesis (*Cit, Cdc6, Kif14, and Birc5*), and cytokines and cytokine receptors (*Gzmb, Il17re, Il23r, Il6, Il7, and Il24*) (Fig. 2G).

Analysis of all genes in skin and gene set enrichment analysis (GSEA) of skin revealed a decrease in leukocyte activation involved in the inflammatory response (NES = − 1.982, P = 0.006), keratinization (NES = − 2.1129, P = 0) and the chemokine signaling pathway (NES = − 1.4549, P = 0.0053) (Fig. 2H). Furthermore, in comparison with IMQ-induced psoriasis mice, the downregulation of IL17A in skin tissues after DNT administration was confirmed through real-time PCR (Fig. 2I).

### Transferred DNT suppressed the presence of IL17A^+^ RORC^+^ γδ T cells

**Fig. 3:**
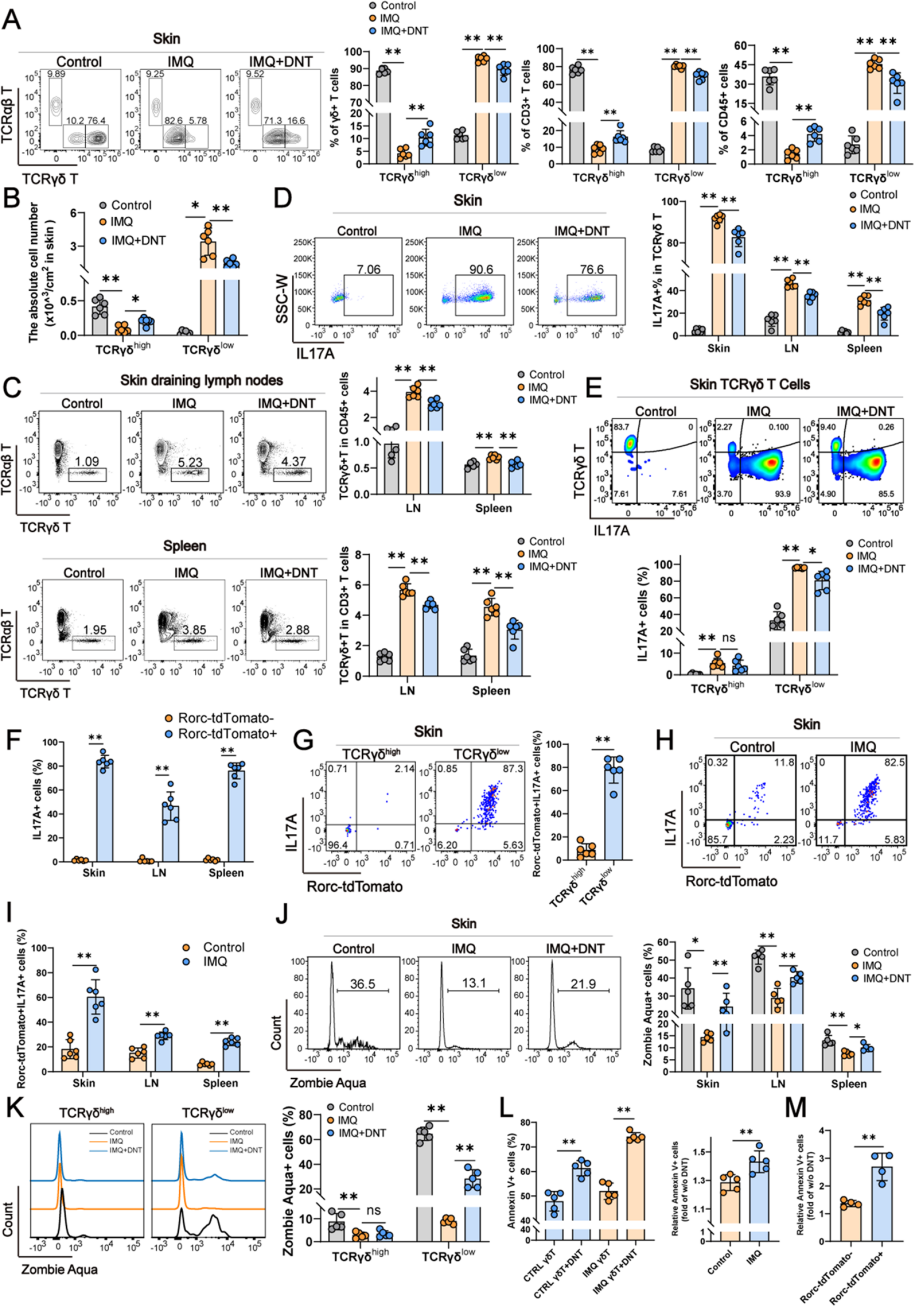
Transferred DNT suppressed the presence of IL17A-producing γδ T cells. **A** Representative flow cytometry plots and statistical analysis of the percentages of skin γδ^high^ T and γδ^low^ T cells relative to the total number of γδ T cells, CD3^+^ T cells and CD45.2^+^ cells in each group. **B** Absolute numbers of γδ^high^ T and γδ^low^ T cells in mouse skin. **C** Representative flow cytometry plots and statistical analysis of the percentages of γδT cells relative to the total number of CD3^+^ T cells and CD45.2^+^ cells in LNs and spleens. **D** Representative flow cytometry plots and statistical analysis of the percentage of IL-17A^+^ cells among skin, LN and spleen γδ T cells. **E** Representative flow cytometry plots and statistical analysis of the percentages of IL-17A^+^ cells among skin γδ^high^ T and γδ^low^ T cells in each group. **F** Statistical analysis of IL17A^+^ cells among Rorc-tdTomato-positive and Rorc-tdTomato-negative γδT cells in the skin, LNs and spleen. **G** Representative flow cytometry plots and statistical analysis of IL17A and tdTomato were measured in skin γδ^high^ T and γδ^low^ T cells by using flow cytometry. **H**, **I** Representative flow cytometry plots and statistical analysis of the percentages of IL17A and tdTomato in the skin, LN and spleen. **J** Representative flow cytometry plots and statistical analysis of the percentages of Zombie Aqua^+^ γδ T cells in the skin, LNs and spleen. **K** Representative flow cytometry plots and statistical analysis of the percentages of Zombie Aqua^+^ cells in skin γδ^high^ T and γδ^low^ T cells. **L** Statistical analysis of the percentages of apoptotic and relative apoptotic γδT cells. **M** Statistical analysis of the percentages of relative apoptotic cells in Rorc-tdTomato-positive or Rorc-tdTomato-negative γδ T cells. Data are presented as the mean ± SD; **A–I** n = 6 mice/group, **J–L** n = 5 mice/group, **M** n = 4 biologically independent samples per group. The normal distribution of variables was assessed using the Shapiro‒Wilk test. One-way ANOVA with a post hoc test for normal variables and the Kruskal‒Wallis test for nonnormal variables were used for multiple comparisons. *p < 0.05, **p < 0.01

Because IL-17-producing γδ T cells are major pathogenic immune cells in IMQ-induced psoriasis, we speculated that the administration of DNT may influence γδ T-cell pro-inflammation in IMQ-induced psoriasis development. Based on the expression levels of TCRγδ, two distinct subsets emerged in the skin: γδ^high^ and γδ^low^ T cells. A typical flow cytometry gating strategy of skin is shown in Additional file 2: Fig. S1A. Upon DNT administration, alterations occurred in the proportions of these γδ T-cell subsets within the skin. The percentage and absolute number of γδ^high^ T cells increased post DNT treatment, while the proportion and absolute number of γδ^low^ T cells decreased (Fig. 3A-B), suggesting that DNT selectively suppressed γδ^low^ T cells but not γδ^high^ T cells. This inhibition of total γδ T cells was also found in the DLNs and spleen (Fig. 3C). However, unlike skin γδ T cells, γδ T cells in the DLNs and spleen did not clearly show γδ^low^ and γδ^high^ subsets by flow cytometry. (Fig. 3C).

γδ T cells mainly secrete IL17A to promote the pathogenesis of psoriasis. We then further investigated the impact of DNT on γδ T-cell IL17A secretion in vivo through flow cytometry analysis. As shown in Fig. 3D, IL17A secreted by γδ T cells significantly decreased after DNT treatment, regardless of the tissue-skin, DLN, or spleen. Intriguingly, we found that γδ^low^ T cells are the major source of IL-17A and that DNT administration downregulates the ability of γδ^low^ T cells to secrete IL17A in the skin of IMQ-treated mice (Fig. 3E), which indicates that DNT inhibits γδ T-cell IL17A secretion mainly through suppression of γδ^low^ T-cell proportions.

Rorc is a crucial transcription factor for IL17A. To further confirm the rationale behind the varying effects of DNT on IL-17-producing γδ T-cell subsets, we developed a transgenic mouse model in which Rorc was labeled with tdTomato fluorescence. In IMQ-induced psoriasis models, IL17A was mainly secreted by Rorc-tdTomato-positive γδ T cells (Fig. 3F). In skin tissue, we also found that Rorc-tdTomato^+^IL17A^+^ γδ T cells were mainly γδ^low^ T cells (Fig. 3G). Compared with the control group, Rorc-tdTomato^+^IL17A^+^ cells also increased significantly in psoriasis mice (Fig. 3H and I).

Furthermore, we explored the manner in which DNT reduces γδ T cells. As displayed in Fig. 3J, IMQ-induced psoriasis displayed a lower death rate of γδ T cells, whereas DNT significantly escalated the death of γδ T cells. Further analysis revealed that DNT chiefly induced death in the γδ^low^ T-cell subset, while DNT did not affect the γδ^high^ T-cell subset (Fig. 3K). We also cocultured DNT with γδ T cells from normal mice and IMQ-treated mice in vitro. As shown in Fig. 3L, DNT induced γδ T-cell apoptosis significantly, especially in IMQ-treated γδ T cells. Meanwhile, in vitro coculture analysis also demonstrated that DNT mainly induced Rorc-tdTomato^+^ γδ T-cell apoptosis (Fig. 3M). The above observations indicated that DNT treatment mainly downregulated Rorc^+^IL17A^+^ γδ^low^ T-cell survival and ultimately led to a decrease in γδ T-cell proportion and IL17A secretion.

### DNT suppressed IL17A-producing γδ T cells through the NKG2D/NKG2DL axis

**Fig. 4:**
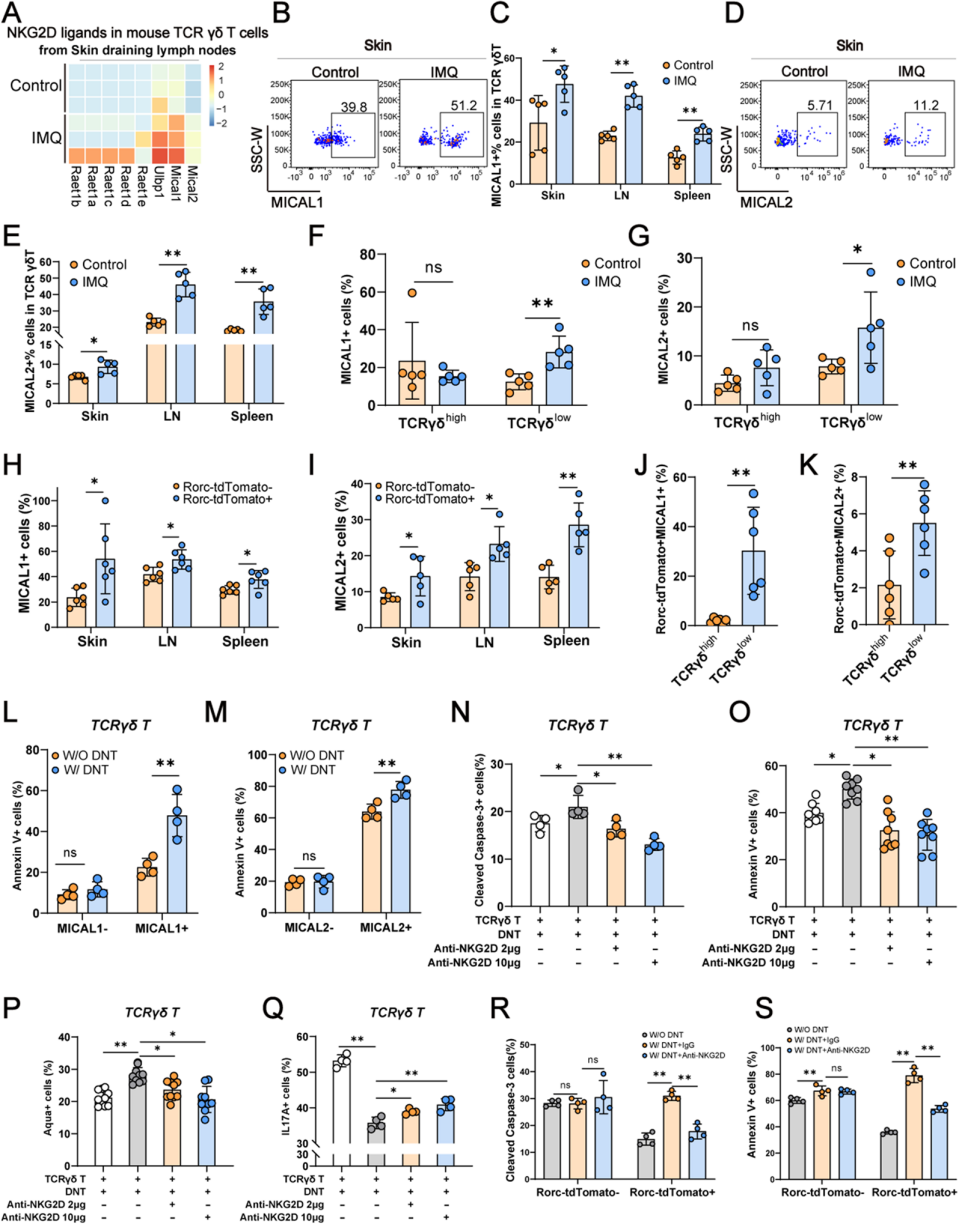
DNT suppressed IL17A-producing γδ T cells through the NKG2D/NKG2DL axis. **A** Heatmap showing genes related to NKG2D ligands in γδ T cells from mouse skin DLNs. **B** and **C** Representative flow cytometry plots and statistical analysis of the percentages of MICAL1^+^ cells in γδ T cells from the skin, LNs and spleen. **D** and **E** Representative flow cytometry plots and statistical analysis of the percentages of MICAL2^+^ cells in γδ T cells from the skin, LNs and spleen. **F** Statistical analysis of the percentages of MICAL1^+^ cells in skin γδ^high^ T and γδ^low^ T cells. **G** Statistical analysis of the percentages of MICAL2^+^ cells in skin γδ^high^ T and γδ^low^ T cells. **H** and **I** Statistical analysis of the percentages of MICAL1/2^+^ in Rorc-tdTomato-positive or Rorc-tdTomato-negative γδ T cells from the skin, LNs and spleen. **J** and **K** Statistical analysis of the percentages of Rorc-tdTomato^+^MICAL1^+^ or Rorc-tdTomato^+^ MICAL2^+^ cells in skin γδ^high^ T and γδ^low^ T cells. **L** and **M** Statistical analysis of the percentages of Annexin V^+^ cells in MICAL1/2-positive or MICAL1/2-negative γδ T cells with or without DNT. **N** Statistical analysis of the percentages of cleaved caspase3^+^ cells in γδ T cells with or without NKG2D blocking antibody. **O** Statistical analysis of the percentages of Annexin V^+^ cells in γδ T cells with or without NKG2D blocking antibody. **P** Statistical analysis of the percentages of Zombie Aqua^+^ cells in γδ T cells with or without NKG2D blocking antibody. **Q** Statistical analysis of the percentages of IL17A^+^ cells in γδ T cells with or without NKG2D blocking antibody. **R** Statistical analysis of the percentages of cleaved caspase3^+^ cells in Rorc-tdTomato-positive or -negative γδ T cells with or without NKG2D blocking antibody. (**S**) Statistical analysis of the percentages of Annexin V^+^ cells in Rorc-tdTomato-positive or -negative γδ T cells with or without NKG2D blocking antibody. Data are presented as the mean ± SD; **A**, **B** n = 3 mice/group, **C–J** n ≥ 5 mice/group, **K–S** n ≥ 4 biologically independent samples per group. The normal distribution of variables was assessed using the Shapiro‒Wilk test. One-way ANOVA with a post hoc test for normal variables and the Kruskal‒Wallis test for nonnormal variables were used for multiple comparisons. *p < 0.05, **p < 0.01

Our previous work characterized γδ T cells from the DLN of mice with IMQ-induced psoriasis by RNA-seq [10]. Further analysis on this RNA-seq showed that NKG2D ligands in γδ T cells were upregulated, especially *Mical1* and *Mical2* (Fig. 4A). Flow cytometry analysis also showed that γδ T cells from IMQ-induced psoriasis mice had higher levels of MICAL1 (Fig. 4B and C) and MICAL2 expression (Fig. 4D and E). Further analysis revealed that the γδ^low^ subset exhibited a significant increase in the expression of MICAL1 and MICAL2 in IMQ-induced psoriasis, while the γδ^high^ subset did not show a notable change in the expression of these ligands (Fig. 4F and G). These phenomena were further confirmed in IMQ-induced psoriasis in Rorc-tdTomato transgenic mice. A higher proportion of Rorc-tdTomato-positive γδ T cells expressed mical1 and mical2 than Rorc-tdTomato-negative γδ T cells (Fig. 4H and I). γδ^low^ T cells had a higher proportion of Rorc-tdTomato^+^NKG2DL^+^ than γδ^high^ T cells in the skin (Fig. 4J and K).

NKG2D is a functional molecule in both human and mouse DNT [13, 17, 18], so we hypothesized that DNT selectively suppresses γδ^low^ T cells through the NKG2D/NKG2DL axis. In line with our hypothesis, DNT primarily induces apoptosis in γδ T cells expressing high levels of NKG2D ligands (Fig. 4L and M). Upon blocking NKG2D, the cytotoxic effect of DNT on γδ T cells was weakened (Fig. 4N-P). Furthermore, we found that DNT can effectively suppress the secretion of IL17A by γδ T cells, when NKG2D was blocked by anti-NKG2D antibody, the secretion of IL17A by γδ T cells was partially restored (Fig. 4Q). Subsequent experimental validation further confirmed that DNT primarily targets tdTomato-positive γδ T cells, and when NKG2D was blocked with an NKG2D antibody, this cytotoxic effect was dampened (Fig. 4R and S). Above all, our findings suggest that DNT selectively suppresses IL17A-producing γδ^low^ T cells through the NKG2D/NKG2DL axis.

### Transferred DNT migrated to skin tissue via CCR5

**Fig. 5:**
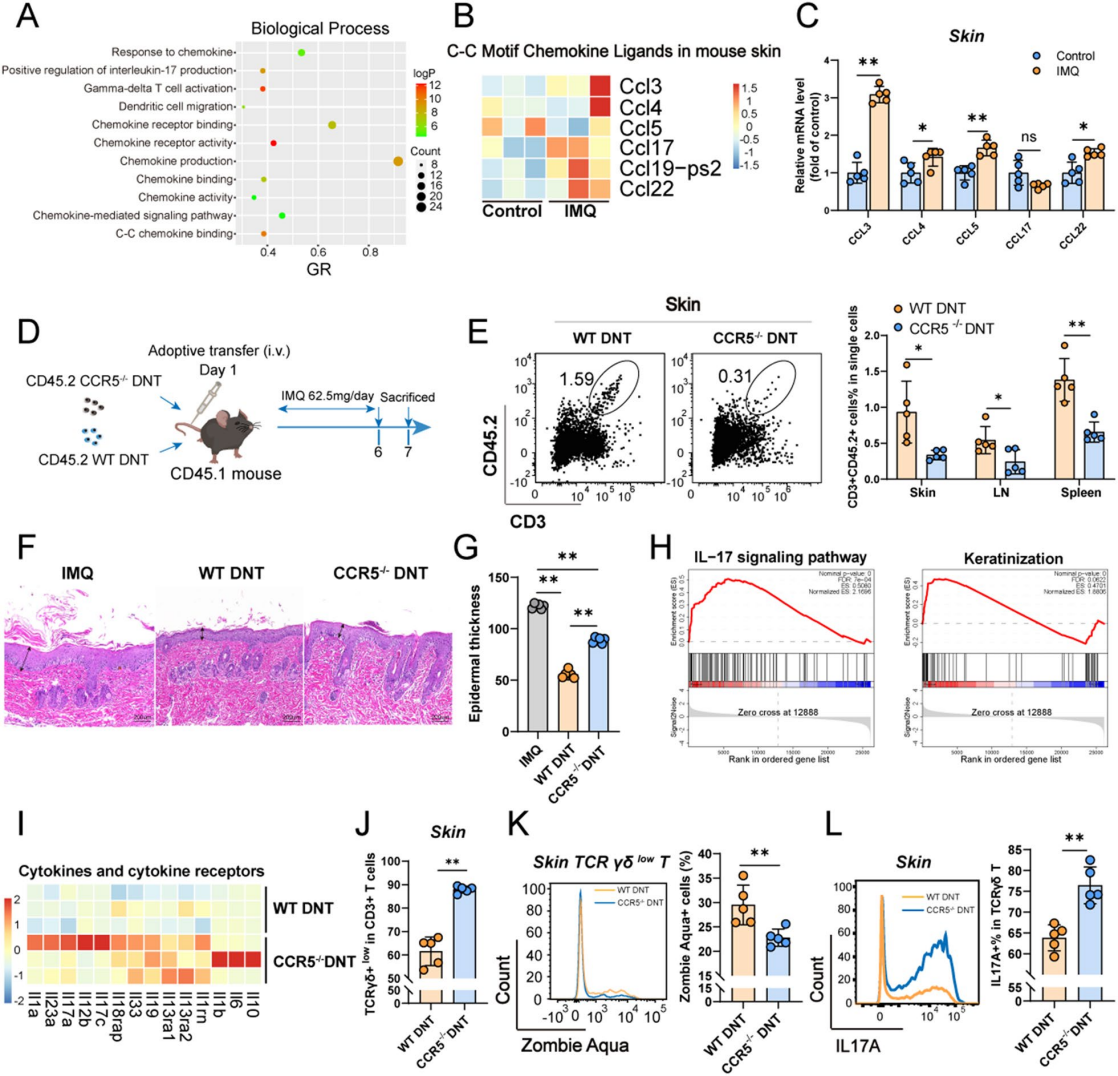
Transferred DNT infiltrate skin tissue via CCR5. **A** Biological process analysis was performed on the basis of DEGs with significantly upregulated and downregulated expression in the skin of IMQ-induced murine psoriasis compared with control mice. **B** Heatmap showing genes related to C–C motif chemokine ligands in mouse skin from IMQ-treated and control mice. **C** mRNA levels of Ccl3, Ccl4, Ccl5, Ccl17, and Ccl22 in mouse skin from IMQ-treated and control mice. **D** Flowchart of Ccr5^−/−^ or WT DNT administration in IMQ-induced psoriasis. **E** Representative flow cytometry plots and statistical analysis of the percentages of transferred DNT (CD45.2^+^CD3^+^ cells in total single cells) that migrated to skin tissue, LNs and spleen. **F** Representative H&E staining of skin sections obtained from IMQ-induced psoriasis and WT or Ccr5^−/−^ DNT-treated mice. The scale bar represents 200 µm. **G** Statistical analysis of the layers of keratinocytes in HE sections obtained from IMQ-induced psoriasis and WT or Ccr5^−/−^ DNT-treated mice. **H** GSEA of the IL17A signaling pathway and keratinization in the skin from Ccr5^−/−^ vs. WT DNT-treated mice. **I** Heatmap showing genes related to cytokines and cytokine receptors in the skin from Ccr5^−/−^ or WT DNT-treated mice. **J** Statistical analysis of the percentages of γδ^low^ T cells in skin CD3^+^ T cells from Ccr5^−/−^ or WT DNT-treated mice. **K** Statistical analysis of the percentages of Zombie Aqua^+^ γδ^low^ T cells in the skin of Ccr5^−/−^ or WT DNT-treated mice. **L** Representative flow cytometry plots and statistical analysis of the percentages of IL17A^+^ cells in skin γδT cells from Ccr5^−/−^ or WT DNT-treated mice. Data are presented as the mean ± SD; n = 5 mice/group. The normal distribution of variables was assessed using the Shapiro‒Wilk test. One-way ANOVA with a post hoc test for normal variables and the Kruskal‒Wallis test for nonnormal variables were used for multiple comparisons. *p < 0.05, **p < 0.01

Previous findings by our group demonstrated that a higher CCR5 was expressed in DNT [19]. To investigate how transferred DNT migrate into skin tissue, we performed transcriptome sequencing on the skin from control and IMQ-treated mice. Gene Ontology (GO) pathway analyses revealed that the differentially expressed genes were involved in the response to chemokines, positive regulation of interleukin-17 production, gamma-delta T-cell activation, chemokines, and chemokine receptor-related activities (Fig. 5A). The heatmap showed that C–C motif chemokine ligands (*Ccl3, Ccl4, Ccl5, Ccl17, Ccl19-Ps2, and Ccl22*) were expressed at higher levels in IMQ-induced psoriasis (Fig. 5B). In addition, real-time PCR of skin tissue showed similar results (Fig. 5C), *and ccl3* expression increased threefold in IMQ-induced psoriasis. WT and *Ccr5*^−/−^ DNT were adoptively transferred into IMQ-induced psoriasis mice (Fig. 5D). Compared to WT DNT, the migration of *Ccr5*^−/−^ DNT was mitigated less in the skin, DLN, and spleen (Fig. 5E). Histological analysis also showed that the mice given *Ccr5*^−/−^ DNT had increased inflammatory cell infiltration and hyperkeratosis compared with WT DNT (Fig. 5F). The layers of keratinocytes in the skin were also increased in *Ccr5*^−/−^ DNT-treated mice (Fig. 5G). Analysis of gene set enrichment analysis (GSEA) of skin tissue RNA sequencing data revealed an increased IL-17 signaling pathway and keratinization (Fig. 5H) in *Ccr5*^−/−^ DNT-treated mice. Furthermore, cytokines and cytokine receptors (*Il1a, Il23a, Il17a, Il12b, Il17c, Il18rap, Il33, Il19, Il13ra1, Il13ra2, Il1rn, Il1b, Il6 and Il10*) were also changed (Fig. 5I). Flow cytometry showed that *Ccr5*^−/−^ DNT had a higher proportion of γδ^low^ T cells in the skin (Fig. 5J). Mice treated *Ccr5*^*−/−*^ with DNT showed less cell death in skin γδ^low^ T cells than WT mice, possibly due to reduced migration of DNT to the skin (Fig. 5K). In addition, *Ccr5*^−/−^ DNT-treated mice exhibited significantly higher secretion of IL17A by skin γδ T cells than WT DNT-treated mice (Fig. 5L). Together, these data demonstrated that transferred DNT migrates to the skin of psoriasis through CCR5 and inhibits IL-17A-producing γδ T cells.

## Discussion

Currently, although biologics targeting TNF-α, IL-12/IL-23 or IL-17A are highly effective in the treatment of psoriasis, not all patients respond in the same manner. Some people might not respond at all (primary treatment failure) or, far more commonly, have an initial response that is subsequently lost over periods of months to years (secondary failure) [20]. Thus, new strategies for psoriasis treatment are still urgently needed.

Increased evidence has indicated the crucial role of DNT in the suppression of CD4 and CD8 T cells [14, 21, 22], B cells [23], NK cells [24], dendritic cells [25] and macrophages [13]. In the present study, we provided evidence that adoptive transfer of DNT migrated to the skin, selectively inhibited IL-17-producing γδ T cells and prevented the development and progression of psoriasis.

The γδ T cells are heterogeneous with multifunctional capacities. Based on the TCRγδ repertoire, γδ T cells are defined as different subsets. Among them, human Vδ1, Vδ2, and Vδ3, murine Vγ2, Vγ4, Vγ5 and Vγ6 γδ T-cell subsets can produce IL-17 in different tissues and diseases [6]. Meanwhile, γδ T cells were also identified as TCRγδ^high^ and TCRγδ^low^ (or TCRγδ^intermdiate^) subsets in both humans and mice based on the intensity of TCRγδ antibody staining evaluated by flow cytometry [9, 26–29]. In a mouse psoriasis model, epidermal T cells were exclusively γδ T cells with high intensity of CD3 and TCR γδ staining and did not produce IL-17 in response to IL-23 stimulation. In contrast, the majority of γδ dermal T cells show intermediate intensity CD3 and γδ TCR staining, and IL-17 is mainly secreted by dermal γδ T cells upon IL-23 stimulation [9].

In our study, we also noticed that in skin samples from mice with psoriasis, TCRγδ^low^ cells were the main γδ T-cell subset that had high RORγT transcription levels and produced IL-17A. Most interestingly, although DNT highly express cytotoxicity-related molecules, including perforin and granzyme B, our data revealed that adoptively transferred DNT inhibited only IL-17A-producing γδ^low^ T cells but not γδ^high^ T cells with no IL-17A secretion, thus ameliorating the development of psoriasis. Although in our previous research, we observed that DNT could induce Th17 cell apoptosis and suppress the production of IL17 by Th17 cells in a NASH mouse model [13]. However, whether DNT have cytotoxic effects on ILC3, Th17, Tc17 or other IL-17-producers in psoriasis are need further investigation.

There have been reports showing that DNT selectively inhibit highly expressed Lag3 [15], NKG2A [13] and NKG2D [18]. NKG2D is located on the cell surface, which is responsible for the recognition and removal of infectious pathogens by lymphocytes and participates in the regulation of the functions of NK and T cells [30]. In our research, IL-17A-producing γδ^low^ T cells highly expressed the NKG2D ligand MICAL1/2. Blocking the NKG2D-NKG2DL interaction reduced the killing effect of DNT against γδ^low^ T cells in a dose-dependent manner, indicating that the selective inhibition of DNT on γδ T cells was NKG2D dependent.

Chemokines and their receptors are important in pro- and anti-inflammatory immune cell trafficking into skin tissue and play critical roles in the development and progression of psoriasis. In psoriasis, keratinocytes produce a broad array of chemokines, including CCL2, CCL3, CCL5, CCL17, CCL20, CCL22, CCL27, CXCL1, CXCL8, CXCL9, CXCL10, CXCL11 and CXCL16 [31].

DNT highly express CCR5, which is critical for DNT migration and function [19]. In our study, we also observed significantly increased CCL3/4 (CCR5 ligands) expression in the inflamed skin tissue of mice with psoriasis. Adoptively transferred DNT were mainly recruited in the skin tissue of psoriatic mouse models. However, adoptive transfer of CCR5 KO DNT failed to transfer to inflamed skin tissue and lost the protective function against psoriasis in mice. These results further confirmed that the CCL3/4 and CCR5 interaction was crucial for DNT migration and function.

## Conclusions

**Fig. 6:**
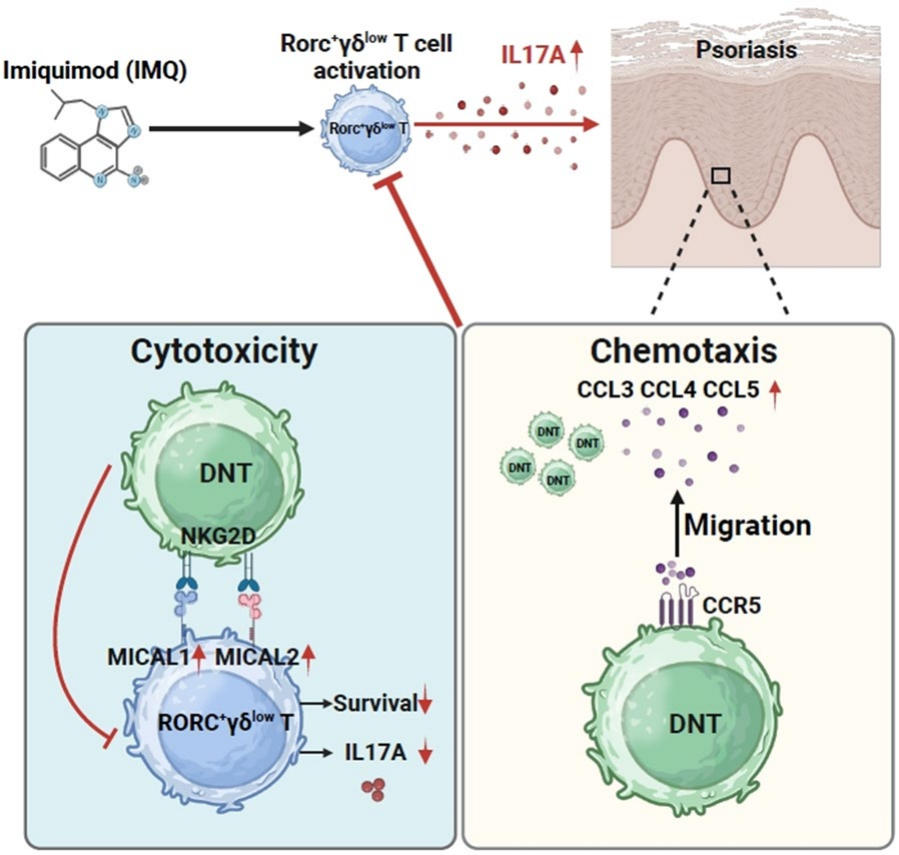
Mechanism of double-negative T cells inhibiting progression against psoriasis. The mechanism of double-negative T cells inhibition progression against psoriasis involved two aspects: cytotoxicity and chemotaxis. In terms of cytotoxicity, within the IMQ-induced psoriasis mouse model, the expression of NKG2D ligands on γδ^low^ T cells in skin was increased, leading to the selectively cytotoxic effect of DNT on γδ^low^ T cells. Regarding chemotaxis, within the IMQ-induced psoriasis mouse model, the increased expression of CCR5 ligands in the skin causes the transfer of DNT to the inflammatory skin area

In this study, we demonstrated that adoptive transfer of double negative cells (DNT) could alleviate the pathogenesis of psoriasis by downregulating the inflammatory response in mouse models. DNT injected from the mouse tail vein could migrate to inflamed skin tissue through CCR5, selectively inhibit IL-17-producing γδ^low^ T cells and finally ameliorate mouse psoriasis (Fig. 6). Our study provides a feasible method for using DNT-based cell therapy for the prevention and treatment of psoriasis in the clinic.

### Supplementary Information

**Additional file 1: Table S1.** Primer sequences used for Real-time PCR.

**Additional file 2: Figure S1.** Representative flow cytometry images of the gating strategy used for flow cytometry analysis. (A) A typical flow cytometry gating strategy in skin.

## Data Availability

The high-throughput sequencing data reported in this work were uploaded in the Gene Expression Omnibus (GEO) database under accession number GSE248626. The following secure token has been created to allow review of record while it remains in private status: qnejsewcdzmhncb.
