# Supplementary figures and images for: Double-negative T cells ameliorate psoriasis by selectively inhibiting IL-17A-producing γδlow T cells
Source: J Transl Med. 2024 Apr 2;22:328. doi: 10.1186/s12967-024-05132-8 (PMC10988838; doi:10.1186/s12967-024-05132-8)

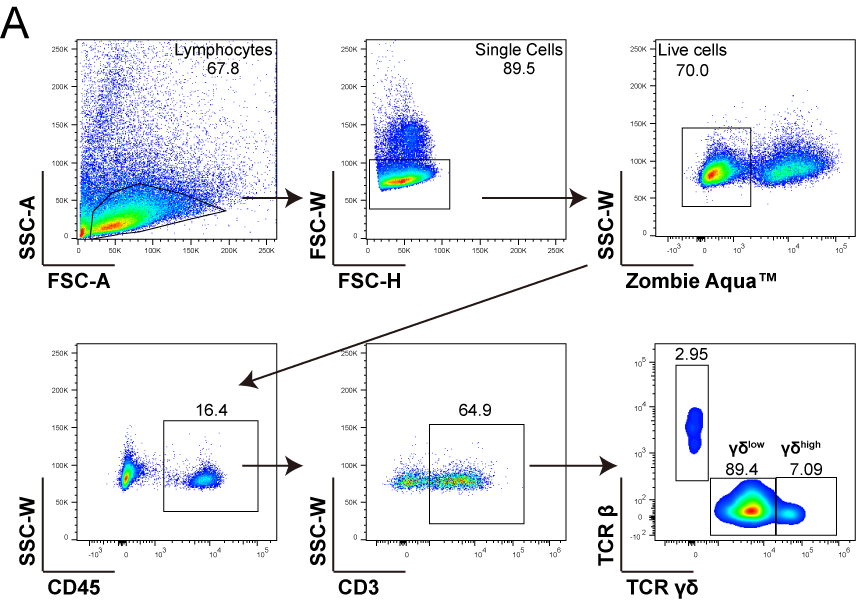

Supplement: Supplementary file 2 — Additional file 2: Figure S1. Representative flow cytometry images of the gating strategy used for flow cytometry analysis. (A) A typical flow cytometry gating strategy in skin. [file 12967_2024_5132_MOESM2_ESM.tif]
